# Editorial

**Published:** 1851-09

**Authors:** 


### THE MEDICAL EXAMINER.

PHILADELPHIA, SEPTEMBER, 1851.

The Medical News and Library, for August, attempts to show that the editorial remarks in our July number, in reference to the action of the National Medical Association upon the report of the Committee on Obstetrics, were predicated upon a misconstruction of the course actually taken. The News gives the following version of the occurrence:

"After the report of the Committee on Obstetrics was read, a delegate stated that some of the statistics referred to in the report were not reliable, and on one or two other points comments were made by other gentlemen. Thereupon the chairman of the committee requested that the report might be referred back to the committee for correction; and, on motion of Dr. Robertson, the report was recommitted. This course was strictly parliamentary, and the Association could not refuse the committee permission to make such alterations as subsequent information induced them to believe to be proper. They were the responsible party, and the judges in the matter, not the Association. The next morning the report was again presented, with some explanations, and with the statement that certain parts had been erased."

We find, however, upon careful examination of the various reports of the *Proceedings*, including that furnished in the June number of the News, that our cotemporary is quite in error in throwing the responsibility of the transaction upon the Committee on Obstetrics. The following extract from the official report of the *Proceedings*, is affirmed by Dr. Robertson, in a pamphlet which he has lately issued, to be the only accurate and authentic report published:

"Dr. Storer, of Ms., Chairman of the Committee on Obstetrics, presented and read the report of the Committee.

"On motion of Dr. Robertson, of South Carolina, the report was recommitted to the Committee, and made the special order for the morning session."

And Dr. Robertson informs us that:

"At the time of making this motion, he stated distinctly, that it was made to give the chairman an opportunity of striking out the statistics of Dr. Ramsay, as it had been asserted, on the most undoubted authority, that they were not reliable."

The motion of Dr. Robertson, therefore, as *adopted* by the Association, was precisely an instruction to the Committee on Obstetrics to strike out from their report statistics which the Association deemed unreliable. We really cannot understand how our cotemporary could assert that "the Association expressed no opinion on the subject."

We are placed, by our cotemporary, in the position of "censuring the Association." This is affixing an application to our remarks which was neither called for nor warranted. And we must beg our cotemporary not to give unnecessary point to our language. We certainly expressed regret at what appeared to have been a hasty proceeding at the Charleston meeting, the full bearing of which could not have been appreciated. And, without intending the slightest disrespect to the Association, we must re-affirm our previous impressions. We cannot see the propriety, or justice, of calling in question the veracity of an absent person, (who had in no way obtruded himself upon the notice of the Association,) upon vague conjectures, and indefinite surmises. And we are satisfied that, even if the statistics in point could have been fairly *proven* to be exaggerated, it would have been far wiser to have avoided this public scandal and altercation. Besides, there are grounds for attributing the movement against our correspondent to pique growing out of a family feud. Personal assaults generally spring from personal animosities, and seldom advance the ends of truth and justice.

---

### MONUMENT TO JENNER.

A Committee, of whom Dr. Conolly, of Hanwell, is Chairman, and George Vere Irving, Esq., Hon. Secretary, has recently been formed, with a view of raising subscriptions for the purpose of erecting a bronze statue of Dr. Jenner, by Wm. Calder Maxwell, A. R. A., to be placed in some public situation in London, as a tribute *from all nations* to the memory of that distinguished philanthropist and eminent benefactor of mankind. With this view, a number of prominent individuals, at home and abroad, have consented to serve on the Committee, and an anxious desire is expressed that the funds of the proposed undertaking "should flow from persons of every station in society, and they, (the committee,) will, therefore, be happy to receive the smallest as well as the most liberal donations."

In our next number we may be able to state further particulars, with the names of those in this country who have consented to serve on the General Committee, and to whom subscriptions may be paid.

---

### OBITUARY.

Died, at Wilmington, N. C., on the 27th of June last, Dr. GEORGE LILLINGTON, aged 24 years.

Dr. Lillington, whose early death is recorded above, was a young man of high promise and brilliant prospects. He fell a victim to a typhoid fever, contracted during fatiguing professional occupations. He was ardent and enthusiastic in the pursuit of knowledge, and warm and generous in his friendships. His preceptor offers this brief tribute to his memory.

